# In silico characterization of ferritin 2 conserved motifs and B-cell epitopes in *Hyalomma anatolicum*

**DOI:** 10.4142/jvs.25152

**Published:** 2026-07-08

**Authors:** Xueqing Zhao, Zeyun Cui, Na Zhou, Yuqian Deng, Licui Wen, Wenying Dang, Caishan Li, Bayinchahan Gailike

**Affiliations:** 1 College of Animal Medicine, Xinjiang Agricultural University, Urumqi 830052, China.; 2 Xinjiang Key Laboratory of Herbivore New Drug Research and Creation, Urumqi 830052, China.; 3 College of Animal Science and Technology, Xinjiang Agricultural Vocational and Technical University, Changji 831100, China.

**Keywords:** Ixodidae, peptide vaccine, immunoinformatics, iron homeostasis, phylogeny

## Abstract

**Importance:**

Ferritin 2 (Fer2) is a well-established candidate antigen for anti-tick vaccines, but few studies have investigated the potential of *Hyalomma anatolicum* Fer2 as an antigenic peptide.

**Objective:**

To characterize the *H. anatolicum* fer 2 coding sequence, conserved motifs, predicted structure, and linear B-cell epitopes.

**Methods:**

The complete coding sequence region of the *H. anatolicum* Fer2 gene was amplified by polymerase chain reaction. The biological properties and effective antigenic peptide of *H. anatolicum* Fer2 protein were analyzed.

**Results:**

The Fer2 sequence of *H. anatolicum* collected showed the highest similarity to that of *H. anatolicum* from Turpan, Xinjiang (accession No. ALJ92580.1), and the phylogenetic analysis accurately classified Fer2 among the different tick species. The biological property results showed that *H. anatolicum* Fer2 is a non-transmembrane protein with a signal peptide and a typical Fer protein structure. Motif analysis showed that different tick 5 motifs were in random combinations, but motifs 1, 2, and 4 were contained in all tick Fer2 proteins. Correspondence existed between motifs and antigens, with motif 1 containing the highest-scoring antigenic epitope.

**Conclusions and Relevance:**

*H. anatolicum* fer 2 motif 1 contains a conserved, high-scoring predicted linear B-cell epitope and warrants experimental immunogenicity and protection studies before consideration as an anti-tick vaccine peptide.

## INTRODUCTION

Ticks (Acari: Ixodidae) transmit a wide variety of pathogens to vertebrates, including viruses, bacteria, protozoa, and helminths [1]. Tick infestations cause stress and immunosuppression in the host, leading to reduced productivity, decreased meat and milk yields, increased morbidity and mortality, and substantial indirect economic losses due to the costs of prevention and control measures. In addition, the associated skin damage reduces its commercial value [2]. Ticks infest approximately one billion cattle globally, leading to annual economic losses estimated at US$14–19 billion in cattle production [3]. Therefore, effective tick prevention and control are critical. *Hyalomma anatolicum* is one of the most widely distributed and economically valuable ticks among arthropods, parasitizing livestock, such as horses, goats, sheep, pigs, and wild animals living in subtropical and tropical regions [4]. A total of 42 tick species belonging to 9 genera have been documented in Xinjiang, accounting for more than one-third of all tick species reported from China [5].

Currently, the misuse of acaricides has led to the development of acaricide resistance and environmental contamination [6]. Therefore, there is an urgent need to develop new strategies to control ticks. Although an anti-tick vaccine is an effective strategy, only the Bm86 vaccine against *R. microplus* is available [7]. The development of the other anti-tick vaccine is still at the stage of vaccine antigen screening. Antigenic peptides have been used in tick control as simple structures and effective vaccine antigen candidates. For example, the immunogenicity of the P0-Bm86 conjugate vaccine against two tick species (*Ixodes ricinus* and *Dermacentor nitens*) was evaluated [8]. The P0-Bm86 conjugate achieved control efficiencies of 63% and 55% against *I. ricinus* and *Dermacentor nitens*, respectively. In addition, animal studies showed that the RmS-17 antigenic peptide markedly reduced egg production and hatchability in Mexican *R. microplus*, resulting in an overall anti-tick efficacy of 79% [9].

Ferritin (Fer) is a highly conserved, multifunctional, multi-subunit biologically active protein found in all living organisms. Fer stores iron, regulates the iron levels, and acts as a detoxifier in case of excess iron [9]. On the other hand, excess ferrous ions have been shown to catalyze the Fenton reaction, generating hydroxyl radicals from hydrogen peroxide that damage cells [10]. Thus far, investigations on tick Fer have continued to advance, with new findings emerging. For example, the recombinant Fer2 protein was used in immunization trials, resulting in cattle producing high antibody titers [11]. Fer2 underwent prokaryotic expression experiments, after which rabbits and cattle were immunized with the recombinant Fer2 antigen [12]. The results revealed a decrease in the number of eggs, weight, and egg production from *I. ricinus* in rabbits, with an overall vaccine efficacy of 98%. In cattle, the vaccine conferred effective protection against *R. microplus* and *R. sanguineus*, with 64% and 72% efficacies, respectively. These findings suggest that Fer2 is a promising candidate antigen for the development of anti-tick vaccines [13].

These studies have shown that Fer2, as an antigen, is effective in controlling the numbers of many different tick species. This study hypothesized that *H. anatolicum* Fer2 could be used as an antigenic peptide for tick control. This study analyzed the genetic evolution and protein characteristics of the complete coding sequence (CDS) of *H. anatolicum* Fer2 and identified the potential structural regions suitable for vaccine development, providing a basis for future Fer2-based vaccines targeting *H. anatolicum*.

## METHODS

### Collection of ticks

The unfed adult ticks were collected in April 2024 from four cattle farms in the Turpan region in Xinjiang, China. The tick specimens were stored in the College of Animal Medicine, Xinjiang Agricultural University, at 25°C with 70% humidity.

### Main reagents

TRIzol reagent and EasyScript One-Step genomic DNA Removal and complementary DNA (cDNA) Synthesis SuperMix (Beijing TransGen Biotechnology, China), and an agarose gel recovery kit (Beijing TransGen Biotechnology) were used in this study. All other routine reagents were of analytical grade, and ultrapure water was used throughout the experiments.

### Main instruments

The polymerase chain reaction (PCR) instrument T100 was purchased from Bio-Rad (USA); the high-speed freezing centrifuge CTR16-2 from Beijing Times Beili Centrifuge (China), the high-speed centrifuge H1650 from Hunan Xiangyi Laboratory Instrument Development (China), and the ultra-clean table SW-SJ-2D from Suzhou Purification Equipment (China).

### Identification of collected ticks

Two hundred eighteen unfed tick samples were identified using the tick morphological identification guide [14]. After DNA extraction, 6 unfed adult males and 6 unfed adult females were selected randomly and identified as *H. anatolicum*. PCR amplification was performed using universal primers for the tick mitochondrial cytochrome c oxidase subunit I (*COI*) gene to identify the tick species. The *COI* primers F (10 µmol/L) (5’-3’): GGTCAACAAATCATAAAGATATTGG, R (10 µmol/L) (5’-3’): TAAACTTCAGGGTGACCAAAAAATCA. The PCR volume was 13 μL; Mix: 6.5 μL, ddH_2_O: 3.5 μL; F/R: 1 μL; DNA (145 ng/µL): 1 μL. PCR reaction condition: 94°C for 5 min, 94°C for 30 sec, 57°C for 30 sec, 72°C for 60 sec, 35 cycles, 72°C for 10 min. Shanghai Sangon Biological Company (China) sequenced PCR products.

### Homology and phylogenetic analysis of the *COI* gene in *H. anatolicum*

Homology analysis of the 12 *COI* gene sequences from *H. anatolicum* was performed using DNAMAN V 6.0. Subsequently, it was analyzed for homology with the *COI* gene from different tick species in the GeneBank database. The genetic evolution of *H. anatolicum COI* was analyzed using the *R. microplus COI* gene (MG721053.1, MG721050.1, and MG721049.1) as an outgroup. The maximum-likelihood phylogenetic tree of the *COI* gene was constructed using IQ-TREE v 2.2.2.6 (model = K2 + G and Bootstrap = 5,000).

### Acquisition of the complete *H. anatolicum* Fer2 CDS

RNA was extracted using the TRIzol method from ticks morphologically identified as healthy. The cDNA was used as a template to amplify the complete CDS region of *H. anatolicum* Fer2 gene with 2 primers, F (10 µmol/L) (5’-3’): ATGGCAGCCACCATGTTTCGA and R (10 µmol/L) (5’-3’): TTACTAGGTGCGCAGTTGCT. The PCR volume was 13 μL; specifically, Mix: 6.5 μL; ddH_2_O: 3.5 μL; F/R: 1 μL; cDNA(163 ng/µL): 1 μL. The PCR reaction conditions were as follows: 94°C for 4 min, 94°C for 30 sec, 57°C for 30 sec, 72°C for 45 sec, 35 cycles, 72°C for 10 min. After the 6 PCR products were purified and cloned into the pMD19-T vector, all of the clones were sequenced by Shanghai Sangon Biological Company.

### Phylogenetic analysis of *H. anatolicum* Fer2

The homology and genetic evolution analyses of the Fer2 gene of *H. anatolicum* were the same as those of the *H. anatolicum COI* gene. The genetic evolutionary analysis model was Q_yeast+G4. Other tick Fer2 protein sequences with high homology to *H. anatolicum* were retrieved from the National Center for Biotechnology Information (NCBI) database, and *Ornithodoros moubata* (accession no. UBR90680.1) was used as the outgroup. All motif analyses of tick Fer2 proteins were performed using the MEME Suite, with the number of motifs set to five.

### Biological characteristics of *H. anatolicum* Fer2

The TMHMM (https://services.healthtech.dtu.dk/services/TMHMM-2.0/) and SignalP-6.0 (https://services.healthtech.dtu.dk/services/SignalP-6.0/) websites were used to predict the transmembrane regions and signal peptides in the *H. anatolicum* Fer2 gene. The B-cell antigenic epitopes and the ab initio protein structure of the Fer2 protein from *H. anatolicum* were predicted using the SVMTriP website and AlphaFold 3, respectively [15]. Visualization of conserved sites was performed using Jelview v2.11.3.3 software. Furthermore, the relationship between the conserved motifs and antigenic epitopes was analyzed using PyMOL v4.6. Briefly, PyMOL was used to identify the conserved motifs corresponding to the antigenic epitopes in the 3-dimensional structure of *H. anatolicum* Fer2. The antigenic epitope with the highest score was selected, and physicochemical property analysis was performed using ExPASy-ProtParam (https://web.expasy.org/protparam/protparam-doc.html).

## RESULTS

### Identification of tick species

*H. anatolicum* exhibited shallow scapular grooves that extended to the posterior margin of the scutum, and the posterior margin of the scutum was smooth. The cervical field depression was apparent. The conscutum was pale coloured. A posteromedial groove is present (it is long and narrow). Leg coloration with indistinct pale rings was observed, but the legs were also pale in a patchy or marbled pattern. The punctuation sizes were small. The punctation distribution was sparse (but with some concentrations of larger punctations at the lateral grooves). The posterior lips of the genital aperture had a broad U shape (alternatively a wide V shape). The subanal plates were aligned with the adanal plates. The subanal plates were indistinct (they were tiny or absent). The adanal plate shape had a round end. The spiracle areas had a sparse setae (Supplementary Fig. 1). The morphological and molecular biology identified the ticks in this morphology as *H. anatolicum*. The specimen was initially identified morphologically as a *H. anatolicum*.

### Molecular identification of *H. anatolicum*

The *COI* gene from all 12 tick samples (PV534868–PV534879) was amplified, producing a 710-bp fragment (Supplementary Fig. 2). Homology analysis showed that the *COI* sequences shared an average identity of 97.13% among the 12 samples. Their sequence similarity with *H. anatolicum* from Pakistan (accession No. MK462201.1) ranged from 99.37% to 99.45%. Except for samples PV534874 and PV534877, all other samples clustered with *H. anatolicum* isolates from Pakistan (accession Nos. MK462201.1, MK462195.1, MK462196.1, and MK462203.1), indicating a close genetic relationship (Fig. 1).

**Fig. 1:**
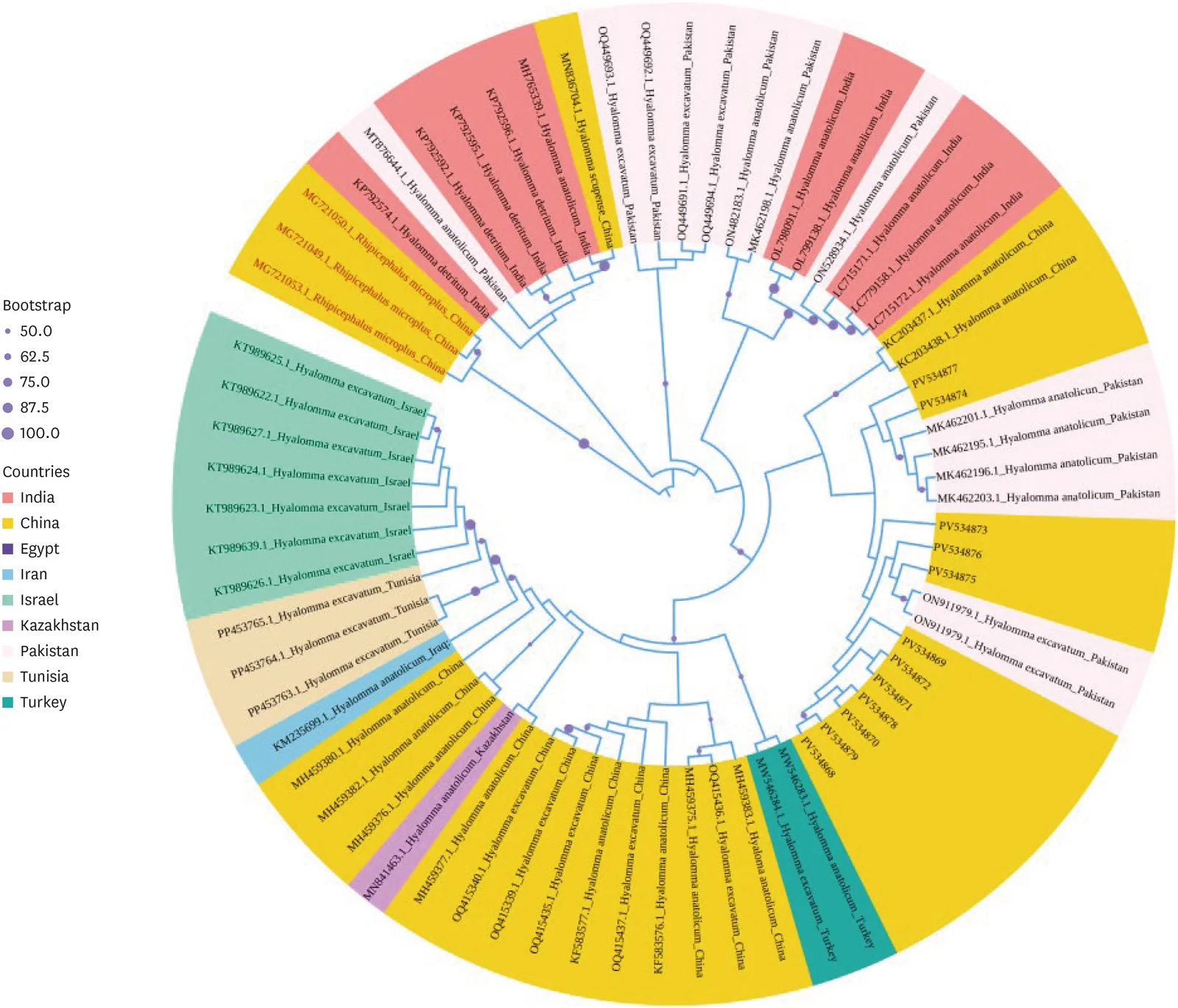
Genetic evolution analysis of the cytochrome c oxidase subunit I gene in *H. anatolicum*.

### Homology and phylogenetic analysis of the ticks Fer2 protein

The Fer2 CDS was amplified in all 6 samples, yielding a fragment of 573 bp in size (Supplementary Fig. 3), NCBI accession numbers: PV536432-PV536437. The 6 samples of the Fer2 gene obtained had 99.43% similarity. Compared with the tick Fer2 in the NCBI database, the results showed the highest similarity of 96.72%–98.95% with the previously NCBI-logged *H. anatolicum* from Turpan, Xinjiang (accession No. ALJ92580.1). The *H. anatolicum* Fer2 amino acid sequences are more closely related to those of *Rhipicephalus* and *Dermacentor*. *Hyalomma* clustering showed that they were closer to *H. rufipes* (Fig. 2A).

**Fig. 2:**
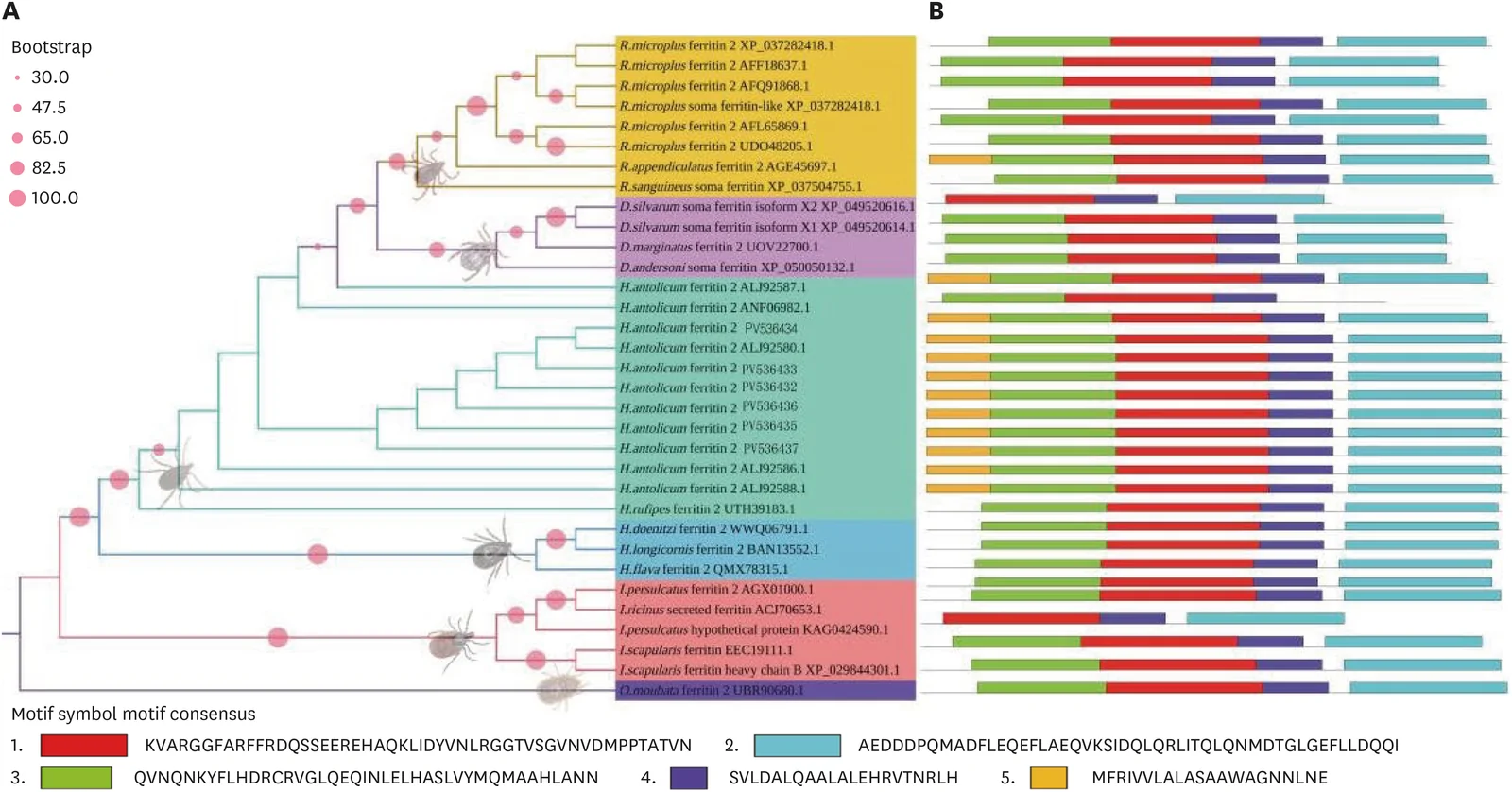
Maximum-likelihood phylogeny and conserved-motif architecture of tick ferritin 2 (Fer2) proteins. (A) Fer2 phylogeny rooted at Ornithodoros moubata (UBR90680.1); circles indicate bootstrap support (Bootstrap 30.0 to 100.0), and colored bands indicate (ticks from different genera). (B) Positions of motifs 1–5 identified by MEME; colors denote motif 1 (red), motif 2 (blue), motif 3 (green), motif 4 (purple), and motif 5 (Orange). Fer2, ferritin 2.

*H. anatolicum* Fer2 obtained in this study, together with the *R. appendiculatus* (accession No. AGE45697.1) and *H. anatolicum* (accession Nos. ALJ92587.1 and ALJ92580.1), all contained five motifs (Fig. 2B). Motif analysis showed that the five motifs (Table 1) existed in different combinations of forms in different tick species Fer2 (Fig. 2B). On the other hand, they all contained motifs 1, 2, and 4 (Fig. 2B), suggesting that these 3 motifs are essential in the chosen tick Fer2 proteins.

**Table 1:** Results of ferritin 2 protein motif analysis of *H. anatolicum*

Motif No.	Amino acid region	Motif amino acid information
Motif 5	1–21	MFRIVVLALASAAWAGNNLNE
Motif 3	26–62	QVNQNKYFLHDRCRVGLQEQINLELHASLVYMQMAAHLANN
Motif 1	63–112	KVARGGFARFFRDQSSEEREHAQKLIDYVNLRGGTVSGVNVDMPPTATWM
Motif 4	113–133	SVLDALQAALALEHRVTNRLH
Motif 2	139–188	AEDDDPQMADFLEQEFLAEQVKSIDQLQRLITQLQNMDTGLGEFLLDQQL

### Bioinformatics analysis of *H. anatolicum* Fer2 protein

The results predicted by TMHMM with Signal-6.0 software showed that the Fer2 of *H. anatolicum* was a non-transmembrane protein with a signal peptide. AlphaFold 3 predicted the complete 3-dimensional structure of the *H. anatolicum* Fer2 protein in this study. An analysis of conserved sites using Jelview v 2.11.3.3 software showed that, compared to other tick species, *R. microplus* and *R. appendiculatus* each harbored a single gene locus mutation, specifically Q86R and V99M, respectively (Fig. 3). The predicted Ramachandran plot of the Fer2 protein model of *H. anatolicum* showed that the amino acid residues fell in 97.2% of the most favored regions and 0% of the disallowed regions (Fig. 4A), suggesting that the established Fer2 protein model of *H. anatolicum* was reliable. Visualization of the three-dimensional (3D) structure of *H. anatolicum* Fer2 protein suggested that it consists mainly of 6 segments of α-helices and a small number of irregularly *COI* led structures (Fig. 4B); 4 nearly perpendicular α-helices formed a lumenal structure (Fig. 4C).

**Fig. 3:**
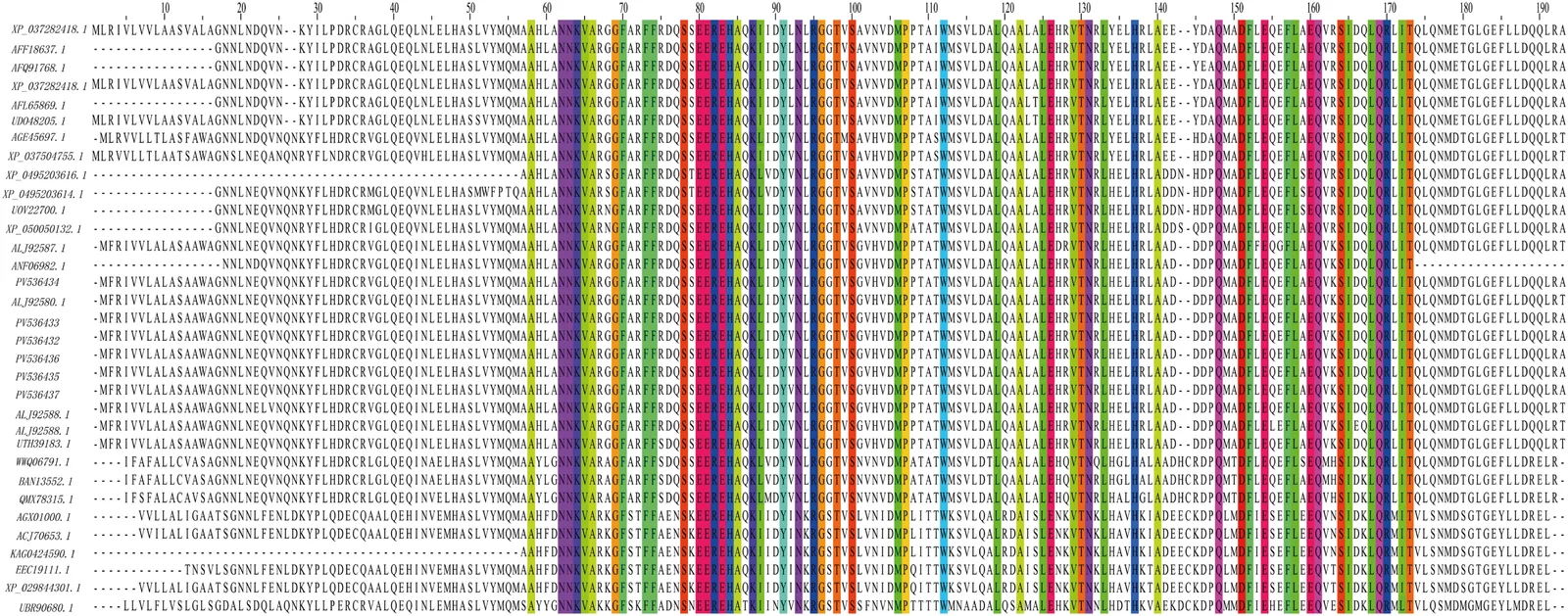
Multiple sequence alignment information of ferritin 2 for different tick species.

**Fig. 4:**
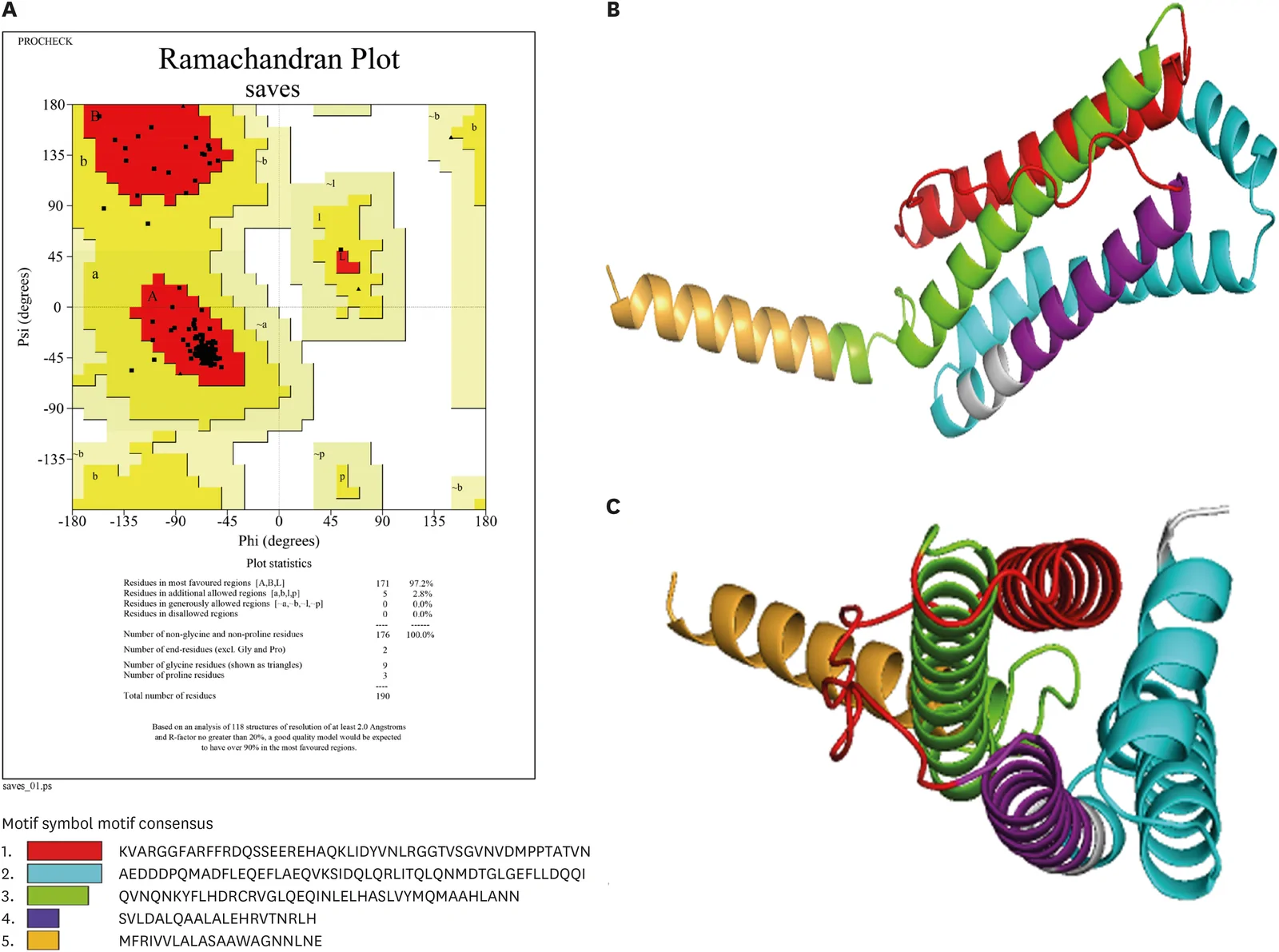
Relationship between the structure of the Fer2 protein and the position of conserved motifs in *H. anatolicum*. (A) *H. anatolicum* Fer2 ramachandran plot; (B, C) *H. anatolicum* Fer2 spatial distribution of 5 motifs. Fer2, ferritin 2.

### Relationship between B-cell antigenic epitopes and motifs

B-cell antigenic epitopes of the *H. anatolicum* Fer2 protein were predicted using SVMTriP, which identified 5 distinct epitope regions. These epitopes were then mapped onto the 3-dimensional structure of the Fer2 protein, showing clear correspondence between the predicted B-cell epitopes and the identified motifs (Fig. 4B and C). Specifically, the highest-scoring antigenic epitope (epitope 3) was located within motif 1 (Table 2). This mapping enabled a detailed assessment of the correspondence between the predicted motifs and the antigenic epitopes of the *H. anatolicum* Fer2 protein (Fig. 4B and C, Table 2). Physicochemical analysis was conducted on epitope 3, which exhibited the highest antigenic epitope score. Motif 1 comprised 20 amino acids, with an isoelectric point of 7.2 ± 0.5. The calculated instability index (II) was 34.73, and the grand average of hydropathy was −1.130, indicating that it is a hydrophilic protein.

**Table 2:** Analysis of ferritin 2 B cell antigen epitopes in *H. anatolicum*

Motif	Amino acid region	Peptide information	Score	Antigenic epitope site
Motif 1	78–97	SEEREHAQKLIDYVNLRGGT	1.000	3
Motif 1, Motif 4	109–128	ATWMSVLDALQAALALEHRV	0.717	4
Motif 2, Motif 4	131–150	RLHELHRLAADDDPQMADFL	0.629	5
Motif 3	42–61	INLELHASLVYMQMAAHLAN	0.587	2
Most of Motif 5	4–23	IVVLALASAAWAGNNLNEQV	0.575	1

## DISCUSSION

*H. anatolicum* is one of the dominant tick species, accounting for more than one-third of the total tick species reported in China. In this study, the tick samples collected from Xinjiang were identified as *H. anatolicum* based on morphological examination according to the manual by Walker and on *COI* sequence analysis [14]. The *COI* gene is one of the most conserved protein-coding genes in the animal mitochondrial genome [15] and is used widely for tick identification. Subsequently, the *COI*-based phylogenetic analysis showed a close genetic relationship with *H. excavatum* (accession No. MK462201.1), which has been mainly reported from the Pakistan region. These findings suggest that there is no clear geographical segregation in the distribution of *H. anatolicum* populations.

Ticks rely entirely on host blood for their survival and reproduction. They ingest large quantities of blood, which exposes them to potentially toxic levels of iron. Therefore, ticks must possess protective mechanisms to counteract the detrimental effects of iron overload [14]. Fer2 is a secreted Fer that plays an essential role in transporting iron to the peripheral tissues [16]. In addition, *H. anatolicum* Fer2 has previously shown its potential as a tick vaccine candidate [1]. Therefore, this study investigated the biological characteristics and antigenic epitopes of *H. anatolicum* Fer2.

This study randomly selected 6 samples of *H. anatolicum* to amplify Fer2 CDS, based on morphology and *COI* gene identification results. *H. anatolicum* Fer2 amplified from the samples in this study had the same size as *R. sanguineus* Fer2 amplified by Zeb et al. [17]. *H. anatolicum* Fer2 was analyzed for homology and found to have the highest degree of similarity to *H. anatolicum* from the Turpan area of Xinjiang. Multiple sequence comparisons showed that not all of the tick Fer2 proteins were complete, but they had a common highly conserved position, suggesting that the Fer2 protein is equally highly conserved in the selected, representative tick species of the *Argasidae* and *Ixodidae*. Furthermore, *R. microplus* and *R. appendiculatus* had 1 locus mutation compared to other tick species (Q86R and V99M, respectively). Genetic evolutionary analyses revealed an accurate intergeneric classification of these different tick Fer2 proteins. For example, *H. asiaticum* clusters with *H. rufipes*. Ndekezi et al. [18] investigated the feasibility of using aquaporin-1 from ixodid ticks as a candidate antigenic peptide through a 3D modeling approach. Following their methodology, a similar computational strategy was applied to analyze the *H. anatolicum* Fer2 protein. Specifically, this study examined and characterized the motifs within the Fer-2 protein sequence with high antigenic epitope potential, aiming to identify regions suitable for vaccine development. Next, the *H. anatolicum* Fer2 protein was analyzed using 3D structural modeling to identify the motifs with high antigenic epitope potential for vaccine development, as previously described [19]. The 3D structural analysis showed that *H. anatolicum* Fer2 shares structural similarity with previously reported Fers [20]. In addition, conserved motif analysis showed that among the 5 motifs identified, motif 1 contained the most highly conserved amino acid residues across Fer2 proteins from different tick species. Specifically, Fer2 proteins from various tick species consistently contained motifs 1, 2, and 4, suggesting that these three motifs may play essential roles in ticks. Furthermore, epitope prediction analysis identified 5 antigenic sites (epitopes 1–5) corresponding to conserved motif regions. Among these, epitope 3 within motif 1 showed the highest antigenicity score, suggesting that it may be a promising vaccine candidate.

Epitopes, defined as antigenic determinants within larger proteins, are considered sufficient to elicit appropriate cellular and humoral immune responses [21,22]. Several peptide-based vaccines are currently under development for various infectious diseases [23,24,25]. In addition, previous studies have explored the use of antigenic peptides for tick control, including the immunomodulatory peptide amregulin from *Amblyomma variegatum* and an antigenic subolesin peptide from *R. microplus* [26]. In this study, motif 1 of *H. anatolicum* Fer2 showed conserved characteristics across tick species and relatively high antigenicity, suggesting it may be a promising vaccine candidate. In particular, epitope 3, located within motif 1, shows potential as a peptide-based vaccine candidate. Nevertheless, further immunological studies using the identified peptide will be necessary to validate these in silico predictions and determine its practical efficacy in tick control.

## SUPPLEMENTARY MATERIALS

Supplementary Fig. 1 Partial results of the morphological identification of *H. anatolicum*. CF, cervical fields; CM, conscutum; SG, scapular grooves; SC, scutum; PU, punctation; LC, leg colouration; PG, posteromedium groove; SE, sparse setae; GA, genital aperture posterior lips; AP, adanal plates; SP, subanal plates.

Supplementary Fig. 2 Polymerase chain reaction amplification results of the *COI* genes in 12 unfed adult tick samples.

Supplementary Fig. 3 Polymerase chain reaction amplification results of the Fer2 gene in *H. anatolicum*.
